# Successful Treatment of Paranasal Sinus Metastasis From Renal Cell Carcinoma With Immune Checkpoint Inhibitors and Radiotherapy: A Case Report

**DOI:** 10.1002/iju5.70156

**Published:** 2026-02-19

**Authors:** Shota Yamada, Tomohiro Matsuo, Ayaka Tsuchiyama, Hiromi Nakanishi, Kensuke Mitsunari, Kojiro Ohba, Yasushi Mochizuki, Ryoichi Imamura

**Affiliations:** ^1^ Department of Urology Nagasaki University Graduate School of Biomedical Sciences Nagasaki Japan

**Keywords:** intensity‐modulated radiation therapy, ipilimumab, nivolumab, paranasal sinus metastasis, renal cell carcinoma

## Abstract

**Introduction:**

Renal cell carcinoma (RCC) metastasis to the paranasal sinuses is rare and lacks standard treatment, particularly for unresectable tumors. We report a case of sphenoid sinus metastasis with sarcomatoid differentiation successfully managed with radiation therapy and immunotherapy.

**Case Presentation:**

A 59‐year‐old male who had undergone left radical nephrectomy for RCC 13 years prior presented with acute left eye pain and tinnitus. Contrast‐enhanced computed tomography (CT) revealed a hyper vascular sphenoid sinus mass with osseous destruction. Biopsy confirmed metastatic RCC with sarcomatoid differentiation. Given rapid symptom progression, intensity‐modulated radiation therapy (39 Gy in 13 fractions) was initiated, followed by immunotherapy with nivolumab and ipilimumab. The patient developed complete blindness, which gradually improved during treatment. Serial CT showed a partial response sustained through four cycles. Subsequent 37 cycles of maintenance nivolumab showed persisting tumor reduction.

**Conclusion:**

Radiation with nivolumab and ipilimumab may be effective for unresectable paranasal sinus metastases of RCC.

## Keynote Message

Although metastasis of renal cell carcinoma to the paranasal sinuses, particularly the sphenoid sinus, is exceedingly rare, no established standard of care exists for unresectable disease. Herein, we report a case of sphenoid sinus metastasis with sarcomatoid differentiation successfully managed with radiation therapy and a combination of immune checkpoint inhibition with nivolumab and ipilimumab.

## 1 Introduction

Renal cell carcinoma (RCC) metastasis to the paranasal sinuses is very rare [1]. However, unresectable tumors present substantial therapeutic challenges. Moreover, although systemic therapy has improved over the past decade, with targeted agents and immune checkpoint inhibitors (ICIs) now central to care, the optimal approach for paranasal sinus lesions remains uncertain, especially when rapid symptoms threaten vision or cranial nerves. We report a case of sphenoid sinus metastasis that responded to radiotherapy combined with ICI therapy.

## 2 Case Presentation

**FIGURE 1:**
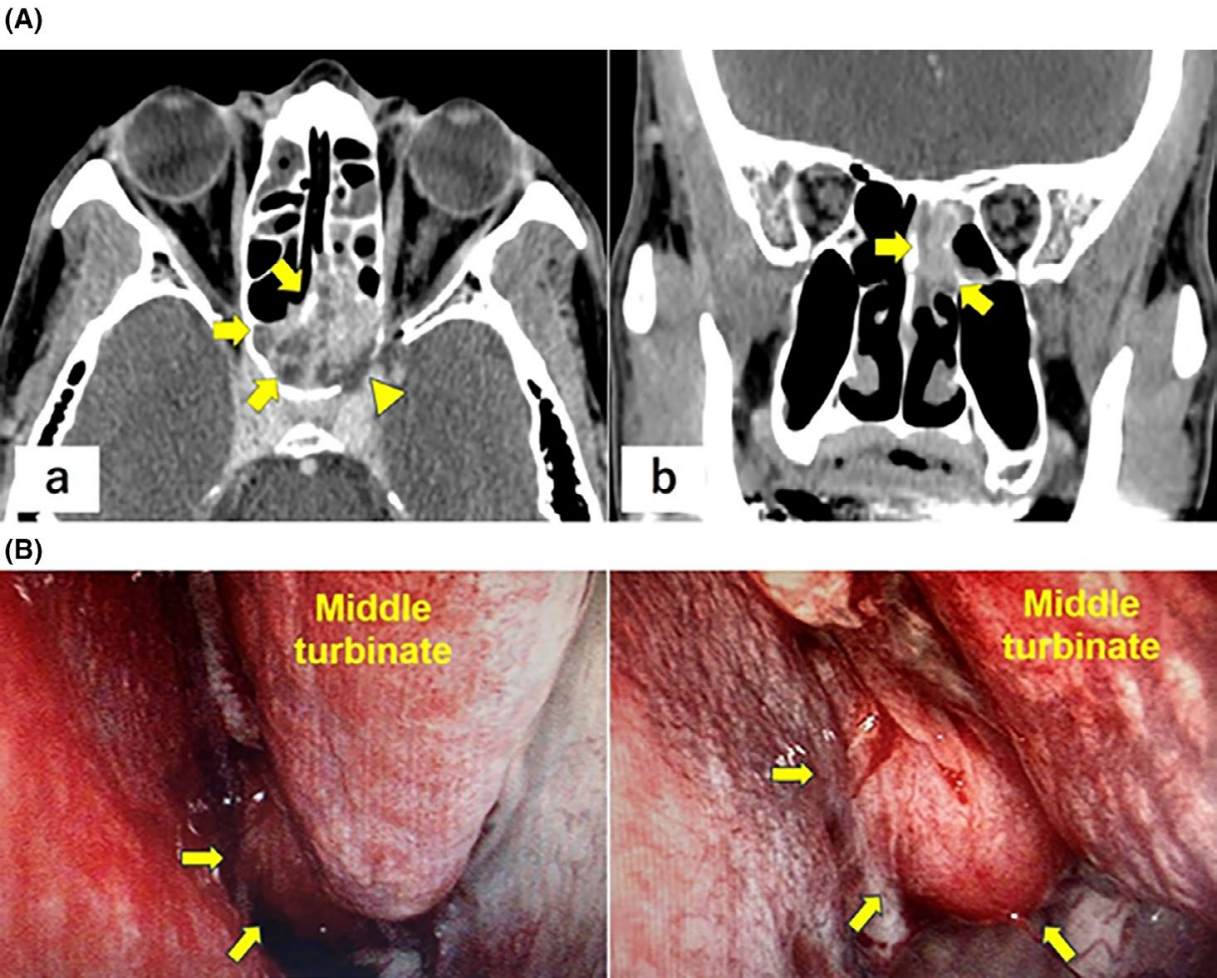
Contrast‐enhanced head computed tomography images at presentation demonstrating an approximately 4‐cm hyper vascular mass centered in the sphenoid sinus with osseous destruction involving the sellar floor and clivus. (A) Axial image. (B) Coronal image. Arrows: Tumors; arrowheads: Osseous destruction. (B) Nasal endoscopic view demonstrating a tumor located posterior to the left middle turbinate. Arrows: tumors. [Correction added on 20 March 2026, after first online publication: Figure 1 and its caption have been corrected to show previously missing bottom panels.]

**FIGURE 2:**
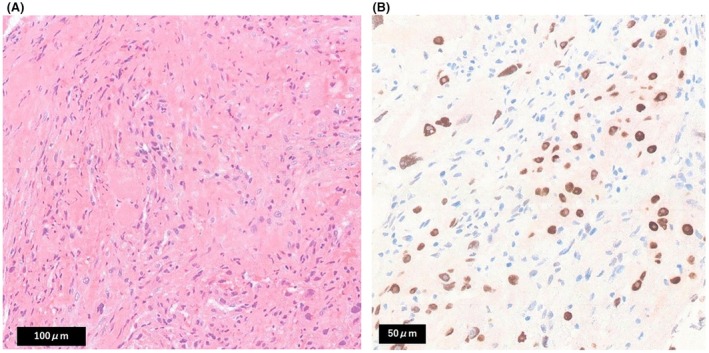
(A) Hematoxylin and eosin–stained section shows atypical spindle to pleomorphic tumor cells with marked nuclear atypia, consistent with sarcomatoid features. (B) Immunohistochemical staining demonstrates Paired box gene 8 (PAX8) positivity, supporting the diagnosis of metastatic renal cell carcinoma derived from clear cell renal cell carcinoma. [Correction added on 20 March 2026, after first online publication: Figure 2 and its caption have been corrected.]

A 59‐year‐old male patient presented with acute‐onset left eye pain and tinnitus. The medical history included hypertension and a left laparoscopic radical nephrectomy for clear cell RCC (pT1bN0M0, G3 < G2, INFα, v0) 13 years earlier. Routine non‐contrast chest/abdominal computed tomography (CT) showed no recurrence, whereas contrast‐enhanced head CT revealed an approximately 4‐cm hyper vascular mass centered in the sphenoid sinus with osseous destruction of the sella turcica and clivus (Figure 1A). [Correction added on 20 March 2026, after first online publication: Figure 1B was deleted in the preceding sentence.] An endoscopic transnasal biopsy was performed (Figure 1B); however, the symptoms rapidly worsened, necessitating urgent admission. [Correction added on 20 March 2026, after first online publication: Figure 1B was added in the preceding sentence.] Repeat CT showed progressive sphenoid destruction and invasion of the left optic canal. Histopathological examination confirmed the presence of metastatic RCC with sarcomatoid differentiation. (Figure 2A). [Correction added on 20 March 2026, after first online publication: Figure  2A has been added in the preceding sentence.] Immunohistochemistry showed Paired box gene 8 (PAX8) positivity, supporting the diagnosis of metastatic renal cell carcinoma (Figure 2). [Correction added on 20 March 2026, after first online publication: The preceding sentence has been added.]

**FIGURE 3:**
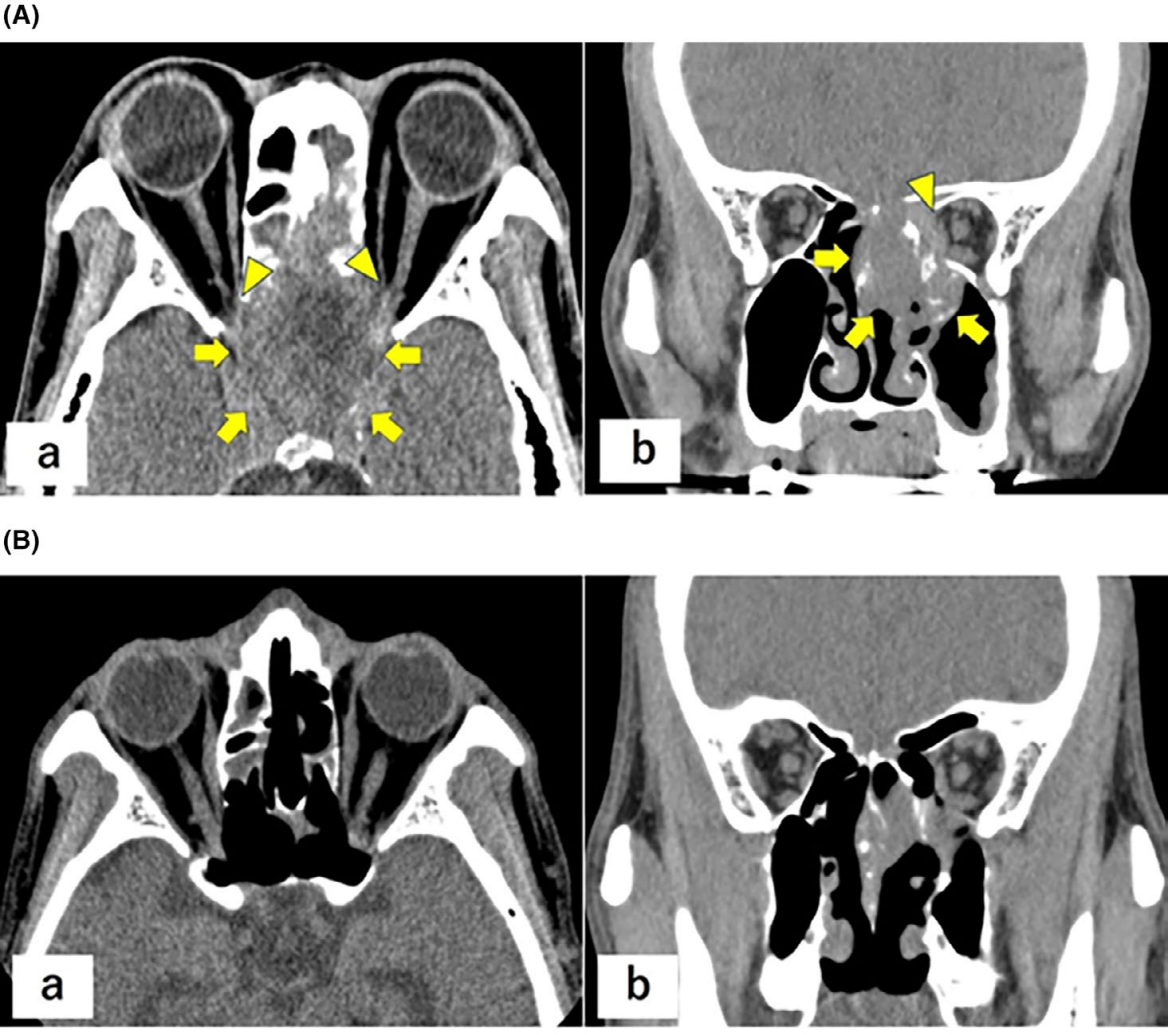
Non‐contrast head computed tomography images showing interval enlargement of the sphenoid sinus tumor with involvement of both optic canals, consistent with optic pathway compromise. (a) Axial image. (b) Coronal image. Arrows: tumor; arrowheads: optic canals. Non‐contrast head computed tomography images during follow‐up demonstrating interval shrinkage of the sphenoid sinus lesion after radiotherapy and immunotherapy, consistent with a partial response. (a) Axial image. (b) Coronal image. [Correction added on 20 March 2026, after first online publication: Figure 3 and its caption have been corrected.]

Intensity‐modulated radiotherapy (IMRT; 39 Gy in 13 fractions) was administered. Upon completion, the patient's visual acuity had declined to light perception only. Given the ongoing deterioration and poor International Metastatic Renal Cell Carcinoma Database Consortium (IMDC) risk (Karnofsky 40%, thrombocytosis 429 × 10^3^/μL, corrected calcium 10.6 mg/dL), nivolumab (240 mg flat dose) and ipilimumab (1 mg/kg) were initiated. On day 3 of treatment, vision showed no light perception, with CT showing evidence of bilateral optic nerve invasion (Figure 3A). [Correction added on 20 March 2026, after first online publication: ‘Figure 2A,B’ has been corrected to ‘Figure 3A’ in the preceding sentence.] From day 20, visual function gradually improved. Visual acuity in the right eye recovered to a level sufficient for independent ambulation, while vision in the left eye returned to light perception. [Correction added on 20 March 2026, after first online publication: The preceding sentence has been added.] Follow‐up CT showed no further growth. The patient was discharged after the second cycle of treatment. CT in the second and fourth cycles revealed tumor reduction (Figure 3B), consistent with a partial response. [Correction added on 20 March 2026, after first online publication: ‘Figure 3A,B’ has been corrected to Figure 3B in the preceding sentence.] After four cycles of nivolumab plus ipilimumab, the patient was transitioned to maintenance nivolumab and completed 37 cycles. The partial response has been maintained, and the patient remains alive 3.5 years after treatment initiation.

## 3 Discussion

Metastatic RCC involving the paranasal sinuses is rare. Metastases occur in the head and neck in approximately 3.3% of cases, with the paranasal sinuses, larynx, and mandible being the most frequent subsites [1, 2]. Potential routes of spread include Batson's valveless vertebral venous plexus, which may permit craniofacial seeding while bypassing pulmonary filtration [3]. Because late recurrence beyond 5 years occurs in approximately 8%–10% of cases, prolonged surveillance remains relevant even after long disease‐free intervals [4, 5]. In our case, a 59‐year‐old male developed a hyper vascular sphenoid sinus mass 13 years after nephrectomy, presenting with acute ocular pain and rapid visual decline, consistent with the anatomic vulnerability of the sphenoid sinus and the adjacent optic apparatus. To contextualize clinical features, treatments, and outcomes in the literature, we summarized previously reported cases (Table 1).

**TABLE 1:** Paranasal sinus RCC metastases, including the present case.

*N*o.	Authors, references	Age/Sex	Time since nephrectomy	Metastatic cites	Systemic therapy	Radiation therapy	Best response	Outcome/follow up
1	Present case	59/M	13 y	Sphenoid sinus	Nivolmab + Ipilimumab	Yes (39 Gy/13 fr)	PR	Alive at 42 months
2	Hess AO et al. [2]	73/M	3 y	Nasal cavity, Ethmoid sinus, Sphenoid sinus, Brain, Adrenal gland	Pembrolizumab	Yes (20 Gy/1 fr)	SD	Alive at 5 months
3	Arai Y et al. [6]	73/M	2 y	Ethmoid sinus	Axitinib	No	PR	Alive at 31 months
4	Parida PK et al. [7]	65/M	15 y	Frontal sinus, Ethmoid sinus, contralateral kidney	Interferon	No	SD	Alive at 2 months
5	Parida PK et al. [7]	58/M	10 y	Ethmoid sinus, Sphenoid sinus, contralateral kidney	Interferon	Yes	SD	Alive at 4 months
6	Parida PK et al. [7]	40/F	12 y	Frontal sinus, Ethmoid sinus, contralateral kidney	Interferon	Yes	SD	Alive at 6 months
7	Marchand‐Crety C et al. [8]	75/M	9 m	Sphenoid sinus	Sunitinib	Yes (27 Gy/3 fr)	CR	Alive at 5 months
8	Sawazaki H et al. [9]	58/M	11 y	Maxillary sinus, contralateral kidney	Interferon	Yes (45 Gy/18 fr)	SD	Alive at 3 months
9	Ranjan SK et al. [10]	74/F	0 m	Frontal sinus, Ethmoid sinus	Pazopanib	No	PR	Alive at 6 months
10	Singh J et al. [11]	48/M	0 m	Nasal cavity	Sunitinib	No	SD	Alive at 3 months
11	Kumar R et al. [12]	42/M	10 y	Frontal sinus, lung	Sunitinib	Yes	SD	Alive at 1 month

Abbreviations: CR, complete response; PR, partial response; RCC, renal cell carcinoma; SD, stable disease.

Epistaxis is the most common presentation, whereas headache, diplopia, and nasal obstruction can also occur depending on the tumor location [2, 13]. Our patient mainly reported ocular pain and tinnitus. Imaging confirmed left optic canal invasion, underscoring the propensity for vision‐threatening progression of sphenoid disease. Diagnosis requires histopathology; in this case, biopsy confirmed metastatic RCC with sarcomatoid differentiation, highlighting the aggressive biology that can drive rapid neurologic compromise and necessitate expedited decision‐making.

When resectable, surgery provides local control. One review reported complete control in 17 of 22 operated cases (77%) [13]. For disseminated or unresectable diseases, care is typically limited to diagnostic biopsy with meticulous attention to bleeding risk, given tumor hypervascularity. Blood loss > 500 mL during sinonasal biopsy has been reported; thus, preoperative embolization may mitigate intraoperative hemorrhage [2, 13]. Our case was deemed unresectable because of skull base invasion and poor risk profile by IMDC, which guided a nonoperative strategy prioritizing urgent local control and early systemic therapy.

Over the past two decades, treatment has shifted from interferon‐based regimens to tyrosine kinase inhibitors, and more recently, ICIs, with case‐level responses to axitinib and a broader adoption of ICIs in metastatic protocols [2, 6, 7]. Radiotherapy, including stereotactic body radiotherapy for sphenoid diseases, is frequently integrated for local control, with favorable responses reported [2, 7, 8, 9]. We administered IMRT (39 Gy in 13 fractions) to the sphenoid lesions. Although vision had declined to no light perception by completion, visual function began to improve on treatment day 20 after initiating nivolumab plus ipilimumab. Serial CT after cycles 2 and 4 demonstrated measurable tumor reduction, consistent with a partial response (Figure 3). These observations parallel reports of sequential modern systemic therapy and focal radiotherapy to balance rapid symptom control with systemic disease management [2, 6, 7, 8, 9, 10, 11, 12]. Our timeline suggests that rapid local debulking with radiotherapy can stabilize the anatomical risk while systemic therapy is mobilized, an approach that may be especially valuable when optic pathway compromise is imminent.

Given the proximity of the paranasal sinuses to the orbits and skull base, progression commonly causes visual impairment and pain, which impacts patient quality of life. Therefore, radiotherapy is a rational adjunct to systemic therapy for unresectable presentations [2, 8, 13]. The emergence of ICIs has renewed interest in their potential abscopal effect, wherein local irradiation augments systemic antitumor immunity [14, 15]. In our patient, the sequence of local radiotherapy followed by ICIs and subsequent systemic radiological improvement paralleled a recent RCC report consistent with the abscopal phenomenon [16]. Although such responses are uncommon, converging evidence indicates that they can occur with ICI exposure and may be potentiated by radiotherapy [14, 15]. Accordingly, our experience supports the careful integration of radiotherapy with systemic immunotherapy, with individualized treatment based on resectability, overall disease burden, anticipated bleeding risk, and urgency to preserve vision or neurological function. In our case, multidisciplinary management led to improved quality of life and comparatively long survival (3.5 years since treatment initiation). Therefore, continuous therapy with regular and careful follow‐up is warranted.

## 4 Conclusion

This case highlights the potential benefits of integrating radiotherapy with ICIs for unresectable sphenoid sinus metastases from RCC. Because this condition is rare, further accumulation of cases is warranted.

## Consent

Written informed consent was obtained.

## Conflicts of Interest

The authors declare no conflicts of interest.

## Data Availability

The data that support the findings of this study are available from the corresponding author upon reasonable request.
