# Correction to “Successful Treatment of Paranasal Sinus Metastasis From Renal Cell Carcinoma With Immune Checkpoint Inhibitors and Radiotherapy: A Case Report”

**DOI:** 10.1002/iju5.70167

**Published:** 2026-03-26

S. Yamada, T. Matsuo, A. Tsuchiyama, et al., “Successful Treatment of Paranasal Sinus Metastasis From Renal Cell Carcinoma With Immune Checkpoint Inhibitors and Radiotherapy: A Case Report,” *IJU Case Reports* 9, no. 2 (2026): e70156, https://doi.org/10.1002/iju5.70156.

An earlier, pre‐revision version of the manuscript was used for publication instead of the final, accepted version. This has resulted in some errors mainly to Figures 1–3, where an incorrect Figure 2 was used and parts of Figures 1 and 3 were missing. Some parts of the text referencing the figures in the Case Presentation section were also affected. The rest of the sections remain unaffected.

In the published version, Figure 1 and its caption were shown as follows:

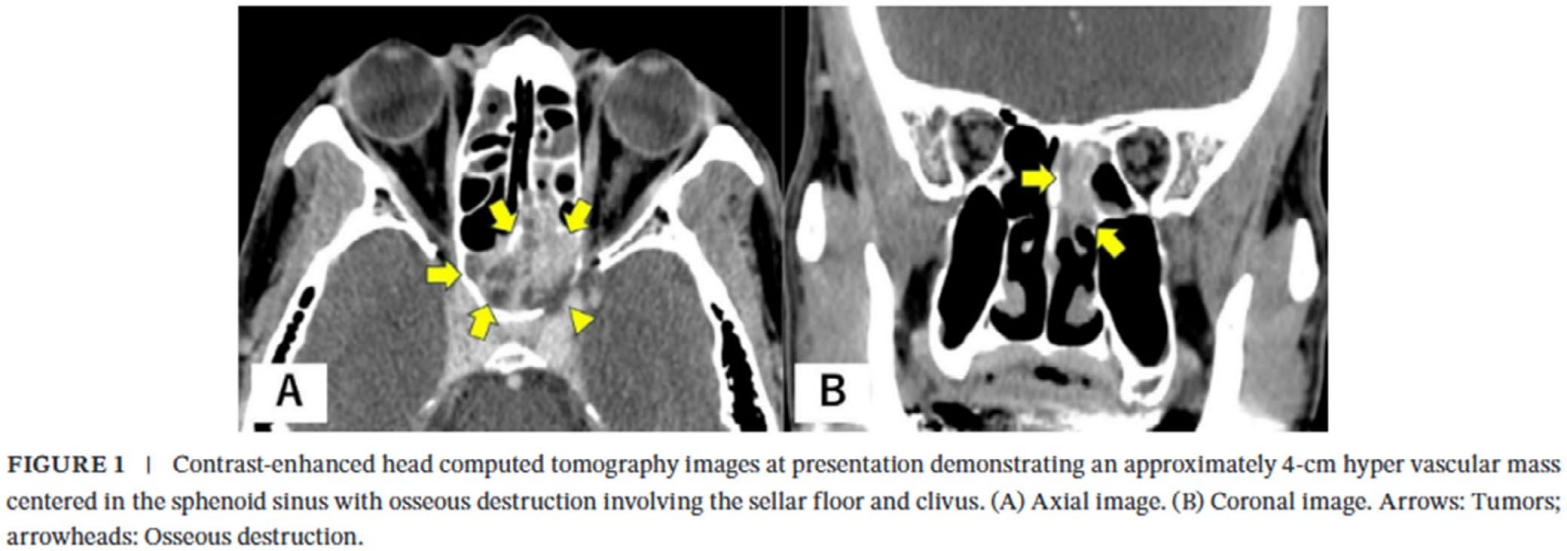

Below are the correct Figure 1 and caption:

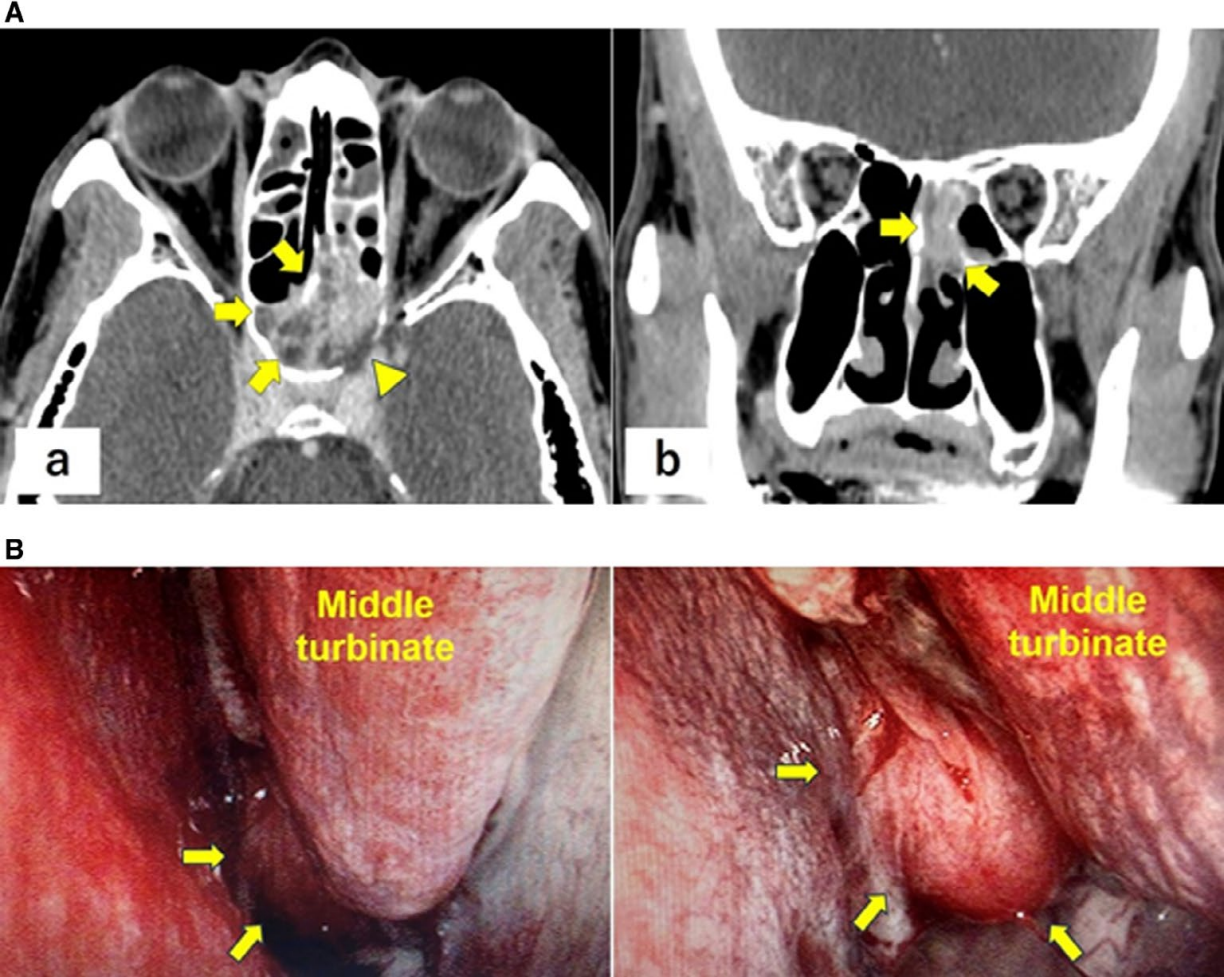

FIGURE 1 (A) Contrast‐enhanced head computed tomography images at presentation demonstrating an approximately 4‐cm hypervascular mass centered in the sphenoid sinus with osseous destruction involving the sellar floor and clivus. (a) Axial image. (b) Coronal image. Arrows: tumors; arrowheads: osseous destruction. (B) Nasal endoscopic view demonstrating a tumor located posterior to the left middle turbinate. Arrows: tumors.

In the published version, Figure 2 and its caption were shown as follows:

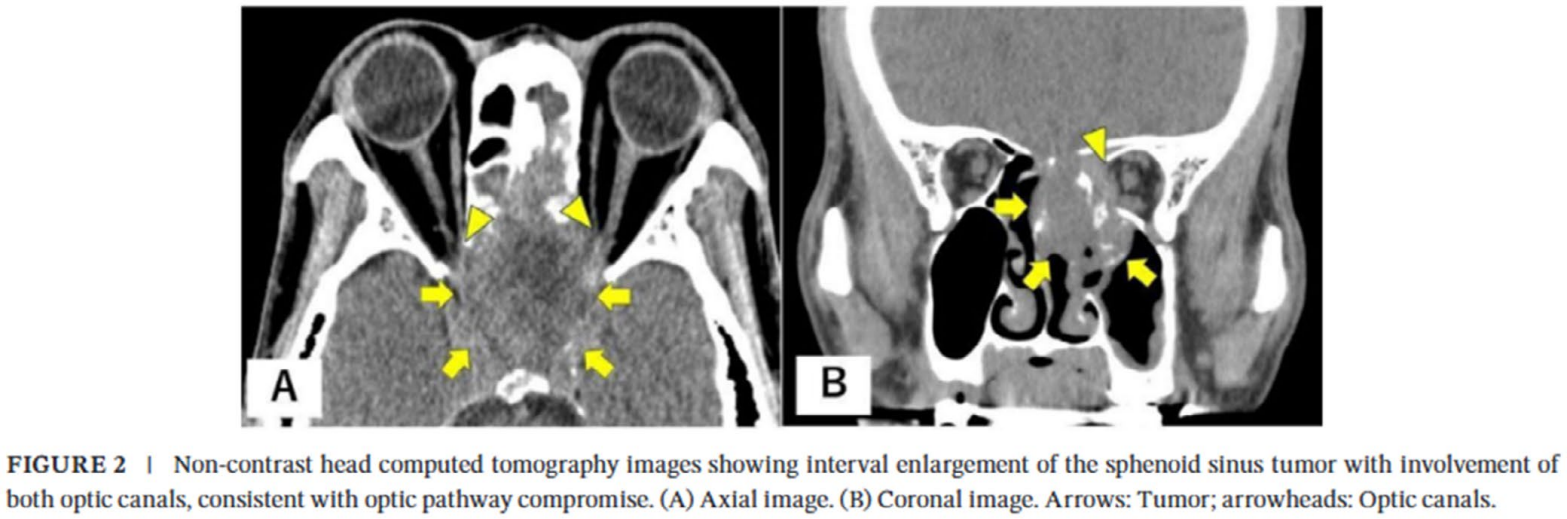

These should be:

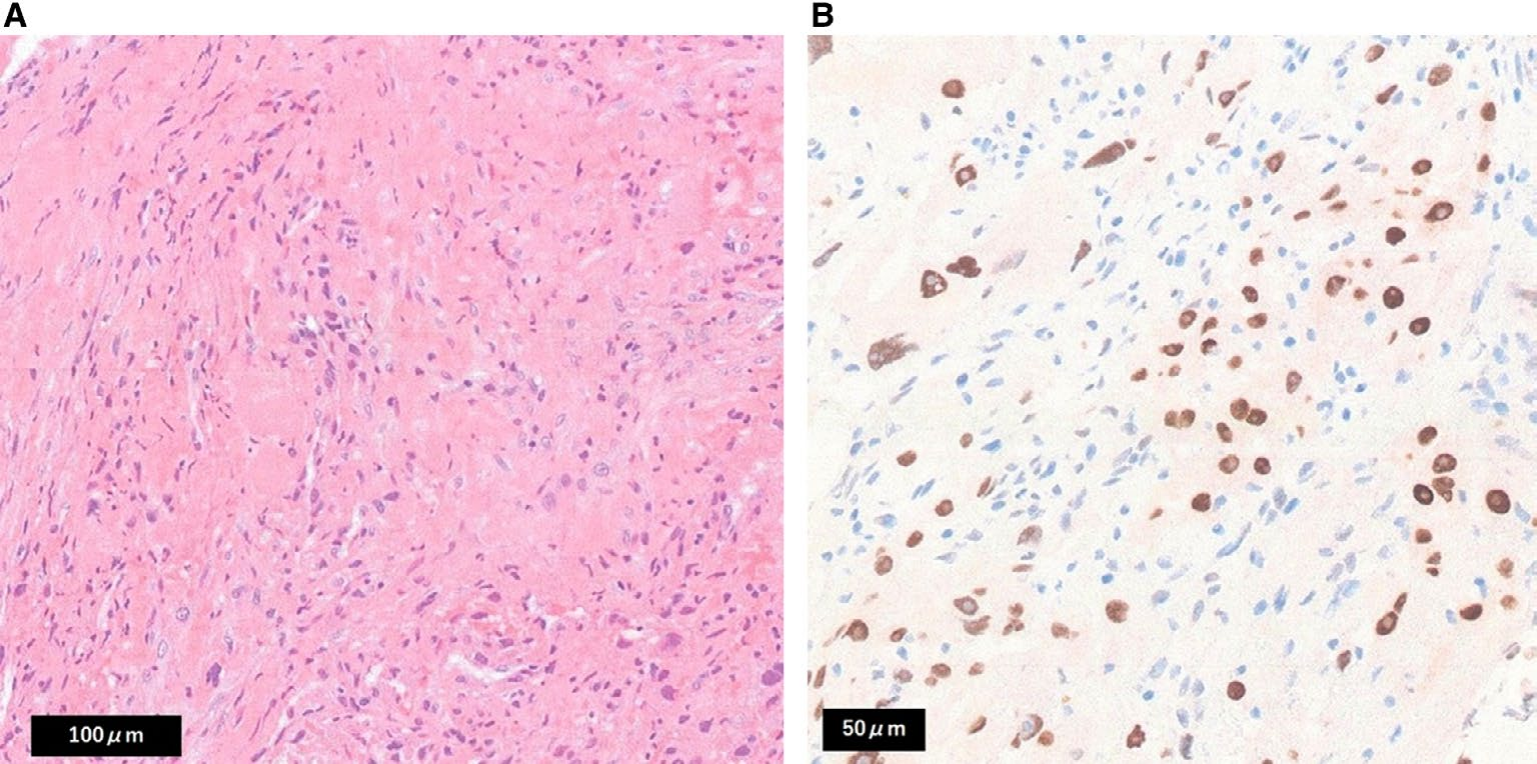

FIGURE 2 (A) Hematoxylin and eosin‐stained section shows atypical spindle to pleomorphic tumor cells with marked nuclear atypia, consistent with sarcomatoid features. (B) Immunohistochemical staining demonstrates Paired box gene 8 (PAX8) positivity, supporting the diagnosis of metastatic renal cell carcinoma derived from clear cell renal cell carcinoma.

In the published version, Figure 3 and its caption were shown as follows:

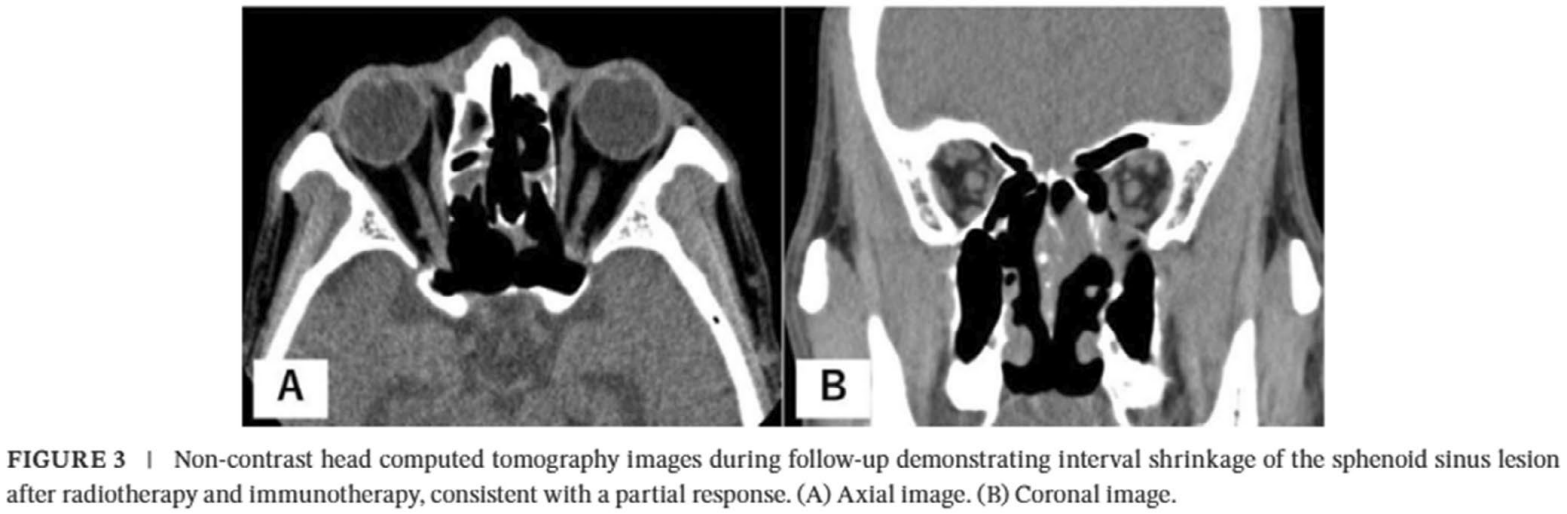

Here are the correct Figure 3 and caption:

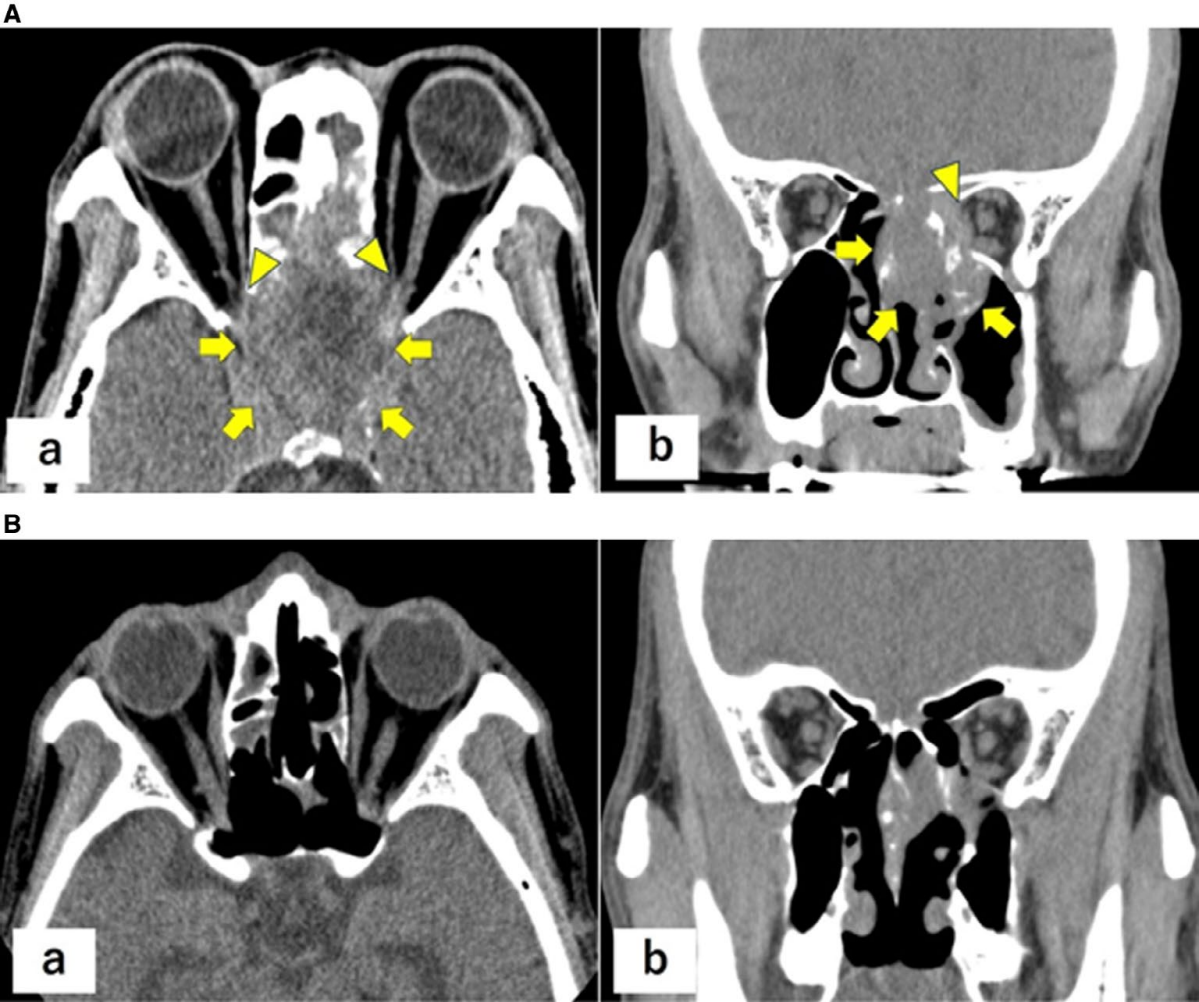

FIGURE 3 (A) Non‐contrast head computed tomography images showing interval enlargement of the sphenoid sinus tumor with involvement of both optic canals, consistent with optic pathway compromise. (a) Axial image. (b) Coronal image. Arrows: tumor; arrowheads: optic canals. (B) Non‐contrast head computed tomography images during follow‐up demonstrating interval shrinkage of the sphenoid sinus lesion after radiotherapy and immunotherapy, consistent with a partial response. (a) Axial image. (b) Coronal image.

The online version of this article has been corrected accordingly.

We apologize for this error.

